# Plant Glutathione Transferases and Light

**DOI:** 10.3389/fpls.2018.01944

**Published:** 2019-01-09

**Authors:** Ágnes Gallé, Zalán Czékus, Krisztina Bela, Edit Horváth, Attila Ördög, Jolán Csiszár, Péter Poór

**Affiliations:** ^1^Department of Plant Biology, Faculty of Science and Informatics University of Szeged, Szeged, Hungary; ^2^Biological Research Centre Institute of Plant Biology, Szeged, Hungary

**Keywords:** circadian regulation, cis-acting elements, dark, glutathione transferase, light

## Abstract

The activity and expression of glutathione transferases (GSTs) depend on several less-known endogenous and well-described exogenous factors, such as the developmental stage, presence, and intensity of different stressors, as well as on the absence or presence and quality of light, which to date have received less attention. In this review, we focus on discussing the role of circadian rhythm, light quality, and intensity in the regulation of plant GSTs. Recent studies demonstrate that diurnal regulation can be recognized in GST activity and gene expression in several plant species. In addition, the content of one of their co-substrates, reduced glutathione (GSH), also shows diurnal changes. Darkness, low light or shade mostly reduces GST activity, while high or excess light significantly elevates both the activity and expression of GSTs and GSH levels. Besides the light-regulated induction and dark inactivation of GSTs, these enzymes can also participate in the signal transduction of visible and UV light. For example, red light may alleviate the harmful effects of pathogens and abiotic stressors by increasing GST activity and expression, as well as GSH content in leaves of different plant species. Based on this knowledge, further research on plants (crops and weeds) or organs and temporal regulation of GST activity and gene expression is necessary for understanding the complex regulation of plant GSTs under various light conditions in order to increase the yield and stress tolerance of plants in the changing environment.

## Introduction

Light is required for optimal plant growth and development, as well as being the most important energy source for biomass production (Chen et al., 2004; Kangasjärvi et al., 2012). At the same time, the presence or absence, period, quality, intensity, and timing of light can alter and influence plant defense responses and induce new signaling and regulation pathways (Chandra-Shekara et al., 2006; Griebel and Zeier, 2008; Ballaré, 2014). Defense responses of plants, especially the induction of locally and systemically acquired resistance or the detoxification mechanism, are significantly regulated by light (Liu et al., 2011; Luschin-Ebengreuth and Zechmann, 2016; Poór et al., 2018). These processes strongly depend on the production and elimination of reactive oxygen species (ROS). Since ROS generation can be influenced by light-driven electron transport chains in the chloroplasts, the production and physiological role of various forms of ROS may differ in illuminated or dark environments (Asada, 2006). Herbicides and other stress factors can decrease and inhibit photosynthetic activity and promote significant ROS generation in plant leaves, thus inducing cell death or several detoxification enzymes implicated in the metabolization of reactive compounds, such as glutathione transferases (GSTs) (Boulahia et al., 2016). However, there is little information about the effects of different stress factors on the expression and activity of many GSTs under various light conditions; furthermore, knowledge on the light-dependent regulation of *GSTs* is still lacking.

The aim of this review was to summarize the current knowledge on the regulation of GSTs in plant developmental processes and stress responses under various light conditions, because these enzymes play a crucial role in the regulation of detoxification processes and homeostasis of ROS. Furthermore, information on the light-dependent molecular regulation of plant GSTs is summarized, which can help to develop innovative procedures in plant protection and crop science depending on light conditions.

## Basic Properties Of Plant GSTs

Plant GSTs (EC 2.5.1.18; GSTs) are a diverse group of multifunctional enzymes, which catalyze a wide range of reactions involving the conjugation of glutathione (GSH; γ-Glu–Cys–Gly) into electrophilic compounds to form more soluble derivatives, which can be transported to the vacuole and further metabolized (Labrou et al., 2015). Plant GSTs consist of three super families (cytosolic, mitochondrial, and microsomal) and can be further divided into distinct classes: tau (U), phi (F), theta (T), zeta (Z), lambda (L), γ-subunit of the eukaryotic translation elongation factor 1B (EF1Bγ), dehydroascorbate reductase (DHAR), metaxin, tetrachlorohydroquinone dehalogenase (TCHQD), Ure2p, microsomal prostaglandin E synthase type 2 (mPGES-2), hemerythrin (GSTH), iota (GSTI), and glutathionyl-hydroquinone reductases (GHRs) (Csiszár et al., 2016).

GSTs represent a relatively large ratio of the total soluble proteins in plant cells, e.g., they comprise ~2% of the soluble protein in wheat seedlings (Pascal and Scalla, 1999). The accumulation of genome sequence data in previous decades revealed several GST homologs organized in complex supergene families in a wide range of plants (Labrou et al., 2015); for instance, in *Arabidopsis thaliana, Solanum lycopersicum, Oryza sativa, Triticum aestivum* there are 55, 81, 83, and 98 members, respectively (Gallé et al., 2009; Dixon and Edwards, 2010; Liu et al., 2013; Csiszár et al., 2014).

Tau and phi classes are the largest groups in plants and play crucial roles in the remediation of environmental pollution by organic xenobiotics, including herbicides, as well as industrial chemicals (Dixon et al., 2003; Benekos et al., 2010; Cicero et al., 2015). Forty-two of the 55 GSTs in *Arabidopsis thaliana* are classified as tau and phi (Dixon and Edwards, 2010; Chronopoulou et al., 2017). Biologically active tau and phi GSTs are dimers and these GST classes are characterized by the presence of a conserved Ser residue at their catalytic site (Nianiou-Obeidat et al., 2017). Tau and phi classes additionally possess glutathione-dependent hydroperoxidase (GPOX) activity in fatty acid hydroperoxides and glutathione conjugation activity in cytotoxic lipid peroxidation products (Nianiou-Obeidat et al., 2017). As they are involved mainly in xenobiotic metabolism, these enzymes possess high affinity for a broad spectrum of harmful compounds, including xenobiotics and endogenous stress metabolites, e.g., lipid peroxides and reactive aldehydes, and may result in high tolerance to abiotic stresses (Gallé et al., 2009; Dixon and Edwards, 2010; Liu et al., 2013; Csiszár et al., 2014). According to detailed studies on safener-induced genome activation, some tau-class GSTs (*AtGSTU19* and *AtGSTU24*) seem to be of significant importance. The induction kinetics of these genes define two classes of xenobiotic response (XR), namely, a rapid (20 min) and a slow (60 min) XR (Skipsey et al., 2011; Brazier-Hicks et al., 2018). The latest results show that a rapid XR is functionally linked to herbicide safening, while testing of oxylipid-inspired safeners differing in their electrophilic properties suggests that differing chemistries result in a distinctive rapid XR (Brazier-Hicks et al., 2018).

Other groups of GSTs have various roles, e.g., participating in hormone signaling or exhibiting peroxidase and isomerase activity (Gallé et al., 2009). The previously mentioned findings about phi- and tau-class GSTs and their ratio to the other members of the GST superfamily also underline their pivotal roles.

At the same time, the complex regulation of GST activity is dependent on the transcriptional and post-translational regulation, which is orchestrated by several promoter elements and transcription factors, and by phosphorylation and S-glutathionylation, which may be dependent on light (Dixon and Edwards, 2010).

## Roles of the Multifaceted Glutathione

Glutathione, the GST co-substrate is synthesized by two ATP-dependent enzymatic steps in the cytosol and chloroplasts (Diaz-Vivancos et al., 2015). First, γ-glutamyl-cysteine is formed by the plastidic glutamate-cysteine ligase, also known as γ-glutamyl-cysteine synthetase (γ-ECS or GSH1), which is the rate-limiting reaction. Glutathione synthetase (GSH2) catalyzes the addition of glycine to γ-glutamyl-cysteine (Noctor et al., 2011). Both *GSH1* and *GSH2* genes respond to light and some stress conditions, such as drought, heavy metals, and certain pathogens (Noctor et al., 2011); thus, GSH may accumulate rapidly under diverse stress effects. It is an essential low-molecular-weight thiol, which fulfills a broad range of functions including as an electron-donating co-factor in biochemical reactions (Noctor and Foyer, 1998; Szalai et al., 2009; Sabetta et al., 2017). GSH is able to control directly or indirectly the level of ROS; thus, it is considered to be one of the most important cellular antioxidants. ROS, such as singlet oxygen (^1^O_2_), the superoxide radical (O${}_{2}^{•-}$), hydrogen peroxide (H_2_O_2_) and the hydroxyl radical (OH^•^), are unavoidable by-products of aerobic metabolism (Foyer and Noctor, 2005). GSH takes part in the removal of the excess amount of H_2_O_2_ as a component of the “Foyer-Halliwell-Asada” or ascorbate-glutathione pathway (Noctor and Foyer, 1998). When GSH reacts with oxidants, it becomes converted into an oxidized form, glutathione disulphide (GSSG).

As a result of the reversible convertibility between the reduced and the oxidized form and the relatively high concentration of the GSH in the cells, glutathione is one of the most important redox buffers. It also represents a storage form of reduced sulfur and can be a signal in the modulation of sulfate uptake and assimilation (Kopriva and Rennenberg, 2004). Being the substrate for phytochelatin synthesis, GSH is a key player in the detoxification of heavy metals (Freeman et al., 2004). As a co-substrate of GSTs, it is involved in the detoxification of different endogenous and exogenous harmful compounds (Cummins et al., 2011). Furthermore, GSH fulfills important roles in the regulation of plant growth, development, and stress tolerance. It is involved in embryo, meristem, and flower primordia development and in pollen germination (Vernoux et al., 2000; Cairns et al., 2006; Gulyás et al., 2014), as well as mediates cell cycle progression and programmed cell death (Kranner et al., 2006; Diaz-Vivancos et al., 2010a,b). In addition, sub-cellular GSH content in leaves of *Arabidopsis* shows a diurnal pattern. The highest content was found after 2–3 h of illumination caused by a strong increase in glutathione synthesis induced by daylight when glycine and cysteine production is restored. In contrast, the lowest GSH content was observed in most cell compartments (mitochondria, plastids, nuclei, peroxisomes, and cytosol) at the end of the dark period, when there was a lack of glutathione precursors, glycine, and cysteine. Thus, GSH content plays a role in the daytime/light-dependent redox balance (Zechmann, 2017).

## GST Expression and Activity are Affected by GSH Concentration and GSH/GSSG Ratio

Plants use oxidants and antioxidants as flexible integrators of signals from metabolism and the environment (Foyer and Noctor, 2013). According to the latest conception, ROS-producing enzymes, antioxidants, and their reduction-oxidation states all contribute to the general redox homeostasis in the plant cell (Potters et al., 2010), but the glutathione has been considered to be the master regulator of intracellular redox homeostasis (Noctor et al., 2011; Foyer and Noctor, 2013). High GSH/GSSG ratio, maintained by increased GSH synthesis and/or GSSG reduction catalyzed by the glutathione reductases (GRs), may provide efficient protection for plants against abiotic stress-induced accumulation of ROS (Szalai et al., 2009).

Characterization of the *Arabidopsis rootmeristemless1* (*rml1*) mutant, which is severely limited in GSH synthesis capacity, revealed that, among the genes regulated by low GSH, 28 GSTs were found (Schnaubelt et al., 2015). Mining of the proteome data for GSH-associated genes showed that disruption of the pathway for the synthesis and degradation of glutathione in the *Atggt1* (γ-glutamyl transpeptidase, which has a function in the degradation of GSH S-conjugates in the vacuole) knockout leaves was associated with the induction of genes encoding four GSTs in the phi class (AtGSTF2, AtGSTF6, AtGSTF9, and AtGSTF10 Ashraf et al., 2010).

Moreover, shifts in the cellular glutathione redox state may reversibly modify redox-sensitive thiol groups in target proteins, either through glutathionylation or formation of cysteine cross bridges. Interestingly, this is even the case for the *Arabidopsis* GSH1 enzyme; thus the synthesis of GSH is under redox regulation. The active enzyme in the oxidized state works as a homodimer linked by two intermolecular disulphide bonds between specific cysteines (Hothorn et al., 2006). As the GSH level increases, in the more reduced intracellular environment, these bonds are disrupted and the enzyme takes on the less-active monomeric form. This post-translational modification provides an efficient and rapid switch mechanism for the control of GSH biosynthesis, ensuring that γ-ECS (GSH1) is activated in parallel with the increased demand for GSH (Jez et al., 2004; Hicks et al., 2007). Furthermore, as a post-translational modification, several GSTs can reversibly be modified by GSH to form disulphides. GSTs containing cysteine in the active site (DHARs, GSTLs, GSTZ1, GSTF7, and GSTU19) and one GST with the ability to form heterodimers with a previously mentioned one (GSTF10 with GSTF7) were proven to undergo S-glutathionylation (Dixon and Edwards, 2010).

## Diurnal Regulation of Plant GSTs

In plants, very important steps of detoxification are catalyzed by cytochrome P450 mono-oxygenases (CYPs) and GSTs. These enzyme systems also contribute to the detoxification of several herbicides, depending on the chemical structure of the herbicide substrate (Cole, 1994; Cummins et al., 1999). Certain herbicides (e.g., triazine, triazinone, and substituted urea) have photo-inhibitory effects by competing with the plastoquinone (PQ) at the QB binding site located on the D1 protein of the PSII complex, causing a high production of ROS and leading to lipid peroxidation and proteolysis of thylakoid membrane proteins, thus inducing cell death (Hess, 2000; Rutherford and Krieger-Liszkay, 2001). Due to their effectiveness in photosynthesis inhibition, they are routinely used for weed control in agro-systems, forests, and roadsides. However, the usefulness of herbicide applications can depend on the light, the photo-inhibitory action of the used herbicide, and the circadian rhythm-regulated defense reaction of plants in the day- or night-time or under different light availability. It is known that phytotoxicity is less prevalent under low light conditions than under strong sunlight (Camargo et al., 2012; Lati et al., 2016; Frenkel et al., 2017). These results also suggest that circadian rhythm and light can be crucial components in these processes, which may determine the effective detoxification of various pollutants or herbicides in plants.

Most organisms do not simply respond to sunrise; rather, they anticipate the dawn and adjust their biology accordingly, as they have the innate ability to measure the time (McClung, 2006). The circadian clock is entrained by light perceived by phytochromes (red and far-red [FR] light receptors), cryptochromes (blue light receptors), and temperature (Greenham and McClung, 2015). Several different clock components with specific peak phases of expression have been described in *Arabidopsis thaliana* (McClung, 2006; Hsu and Harmer, 2014). The endogenous system of the circadian clock allows for the daily adaptation and optimization of plant physiology and metabolism. A major function of the circadian clock was suggested to confer an adaptive advantage by the synchronization of metabolic and physiological processes with environmental changes (Alderete et al., 2018). Moreover, the circadian clock acts as a strategic planner to prime active defense responses, which depend on the cellular redox state (Karapetyan and Dong, 2017). Hence, the disturbance of the circadian clock leads to a number of cellular misregulations, including the downregulation of immune responses (Grundy et al., 2015). In addition, circadian rhythm could have important consequences for physiological outcomes of chemical exposures (e.g., herbicide application) at different times of the day (Hooven et al., 2009). ROS are key components in the signaling of immune response. The production, response, and transcriptional regulation of ROS scavenging genes are controlled by the circadian clock. ROS-dependent genes show time of day-specific expression patterns regulated and coordinated by the core-clock regulator, *Circadian Clock Associated 1* (*CCA1*) (Lai et al., 2012).

Based on the first observations, GSH content showed high concentrations during the midday period and low concentrations during the night in spruce (*Picea abies* L.) needles (Schupp and Rennenberg, 1988) and in Canary Island pine (*Pinus canariensis* C.Sm.) needles (Tausz et al., 2001). Other authors have also confirmed that GSH content was relatively low in the dark phase, but increased by illumination in the light phase in poplar (*Populus tremula* × *Populus alba* L.) leaves (Noctor et al., 1997). GSH slightly increased during the day in tobacco (*Nicotiana sylvestris* Speg. & Comes) leaves (Dutilleul et al., 2003). Huseby et al. (2013) also demonstrated that the first 4 h of exposure to daylight significantly elevated GSH content in the leaves of *Arabidopsis*. Thus, diurnal regulation of GSH takes part in cellular redox control (Zechmann, 2017).

In mammals, it is already well-known that key detoxification enzymes, like GSTs, show strong circadian transcriptional regulation (Abhilash et al., 2009). However, there is little information about the putative circadian regulation of these genes in plants. Alderete et al. (2018) analyzed the putative circadian regulation of genes involved in the metabolism of xenobiotic compounds, such as *NtGST* in tobacco plants. Tobacco (*Nicotiana tabacum* var. Wisconsin) seedlings and tobacco hairy root cultures were synchronized by 12 h of light/12 h of dark and treated with phenol, after which the expression of detoxification enzymes was determined in 2- and 3-weeks-old cultures. In tobacco seedlings, the selected *NtGST* gene (phi class) showed diurnal regulation with increased expression at the end of the light phase, with transcript levels decreasing in the dark period. In 2-weeks-old hairy root cultures, the relative transcript amount of *NtGST* was rather oscillating, while, in 3-weeks-old hairy root cultures, the expression pattern was similar to that in seedlings. Phenol treatment highly affected the expression of *NtGST* as it revealed a trend consisting of downregulation during the day and upregulation during the night (Alderete et al., 2018). Gallé et al. (2018) also found that both the GST activity and expression levels of selected *GSTs* reached the maximum at the end of the light period, before both decreased under darkness in leaves of tomato (*Solanum lycopersicum* L.).

## GST Gene Expression and Enzyme Activity are Affected by Light Quality

Light as one of the most important environmental signals regulates plant development and defense mechanisms throughout the plant life cycle. For plants, the blue and red wavelengths of the light spectrum, which is utilized for photosynthesis, are the most important. Thus, the blue light-sensing cryptochrome (CRY) and red light-absorbing phytochrome (PHY) play important roles in the regulation of plant light responses, such as light-dependent seed germination, de-etiolation, shade avoidance, stomatal development, circadian rhythm, and photoperiodic flowering (Su et al., 2017). However, high light and particularly its integral ultraviolet (UV) part causes stress, potentially leading to serious damage to DNA, proteins and other cellular components (Müller-Xing et al., 2014).

Loyall et al. (2000) were pioneers in the research on *GSTs* transcriptional response to short wavelength light. UV-A, UV-B, and red and blue light-induced genes were identified by fluorescent differential display in parsley (*Petroselinum crispum* (Mill.) Fuss) cell cultures, and it was found that UV-B induced the expression of tau-class *GSTs* (Loyall et al., 2000). This was the first report on UV-B inducibility of *GSTs*. Other regulator signals of the identified *GST* were defined with RNA gel blot analysis. Two-hours-long UV-B and hormone (2,4-D and α-naphthylene acetic acid) treatments resulted in an outstanding induction of *PcGST1* (AF177944) expression in parsley cell culture. The UV-B caused rapid increase of PcGST1 mRNA preceded the induction of chalcone synthase (CHS), which gene product is produced in the vacuoles protecting plants from UV-B irradiation (Müller-Xing et al., 2014). The co-expression of *PcGST1*, together with a *LUC* reporter gene under the control of a *CHS* promoter, resulted in an earlier UV-dependent *CHS*:*LUC* induction. The addition of GSH to the *GST* × *CHS:LUC* cell cultures led to an UV-B-independent elevation of the LUC emission 2 h after the application. This first peak was followed by a peak at 6 h. In brief, Loyall et al. (2000) provided evidence for a novel function of GSTs involved in the UV-B mediated signal transduction to CHS, in which external GSH and *PcGST1* possibly affected the *CHS* transcription by changing the redox state. Further, in a proposed model for UV-B-mediated signal transduction, the changes in the redox state and in GST gene expression were preceded by increased intracellular calcium levels in CHS-specific gene expression (Frohnmeyer and Staiger, 2003). More recently, the induction of plant GST activity and/or gene expression by UV-B, UV-A, or UV-C stress was verified in several other higher plant species: *Brassica rapa* L. (Zhou et al., 2007), *Vitis vinifera* L. (Kobayashi et al., 2010; Pontin et al., 2010), *Brassica oleracea* L. var. italica (Mewis et al., 2012), *Miscanthus sinensis* Andersson (Seong et al., 2015), *Vaccinium corymbosum* L. (Inostroza-Blancheteau et al., 2016), and *Azolla* sp. plants (Prasad et al., 2016).

**Figure 1:**
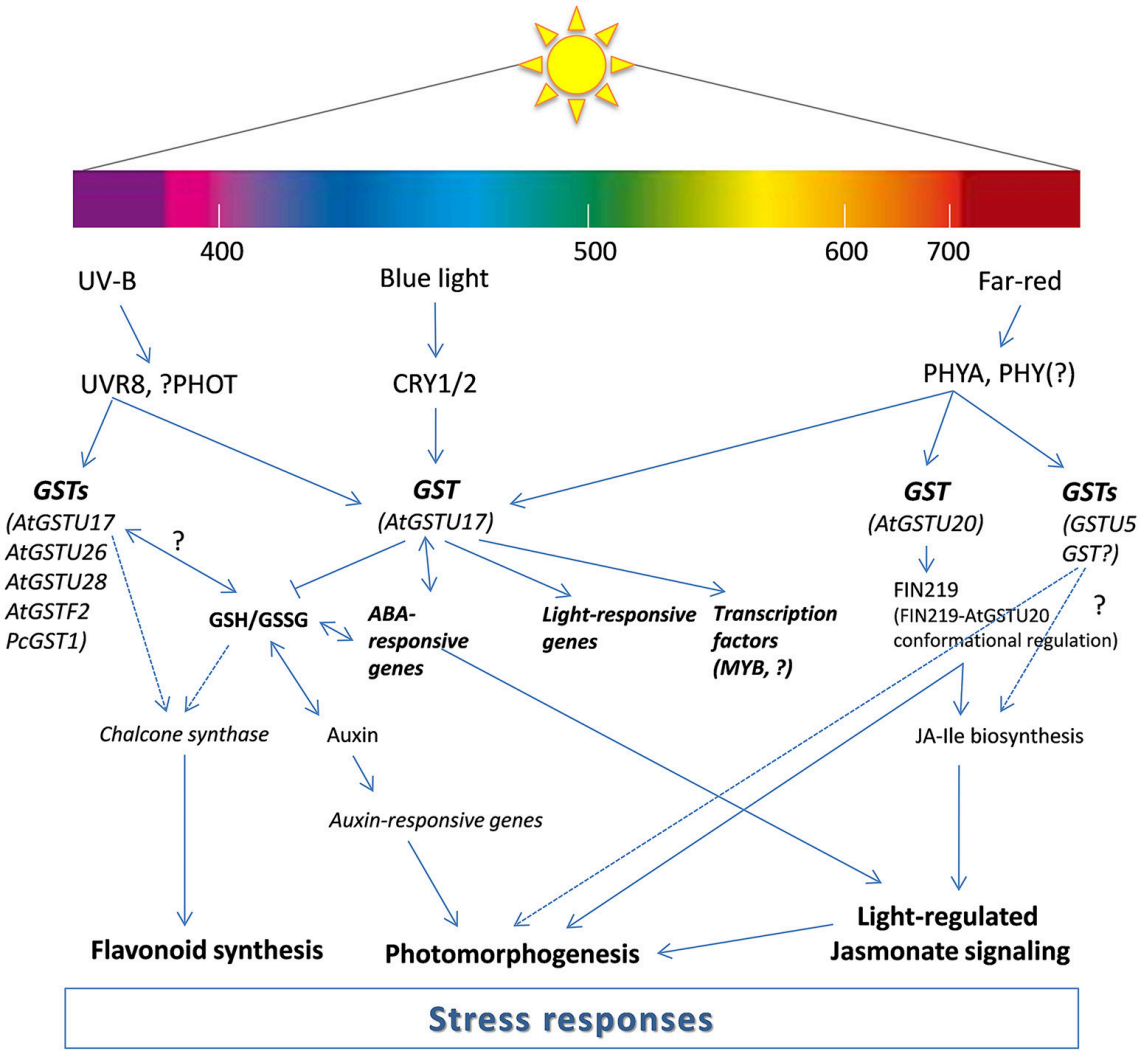
Proposed model for the participation of glutathione transferases (GSTs) in light signal transduction. The model is modified from Frohnmeyer and Staiger (2003), Jiang et al. (2010), Loyall et al. (2000) and Chen et al. (2017). The model illustrates transcriptional and post-transcriptional regulation of GSTs by light (UV-B, blue, and far-red) and possible function of GST proteins in the light induced signaling pathways. *AtGSTU17* was reported to fine tune GSH homeostasis and GSH/GSSG ratio and regulate auxin, ABA, and light response. AtGST20 is having a role in jasmonate (JA) signaling as a conformational regulator of FIN (FR-insensitive 219). Other *GSTs* (*AtGSTU26, ATGSTU28, AtGSTF2*, and *PcGST1*) are also parts of light (UV-B)-regulated signaling which possibly affect chalcone synthase transcription. ABA, abscisic acid; CRY1/2, cryptochrome 1/2; GSH, reduced glutathione; GSSG, oxidized glutatione; JA-Ile, jasmonoyl-isoleucine; MYB, myeloblastosis transcription factors; PHOT, phototropin; PHY, phytochrome; UVR8, UV resistance locus 8.

Red and FR light-absorbing photoreceptors (PHY family) regulate multiple plant growth and developmental responses. Tepperman et al. (2001) firstly observed that the expression of one *Arabidopsis GST* belonging to the tau class (AAD32887) increased rapidly after FR light irradiation, but it was inhibited by phytochrome A (*PHYA*) mutation (Tepperman et al., 2001). Later, Chen et al. (2007) identified that AtGSTU20 interacts with FIN219 (FR-insensitive 219), meaning it is a part of the PHYA-mediated, FR-induced signaling network. Using gain-of-function and loss-of-function mutants, AtGSTU20, also called FIP1 (FIN219–interacting protein) was proven to have a complex function in the regulation of development, as it resulted in a FR-hyposensitive hypocotyl (gain of function) or in a delayed flowering phenotype (loss of function). Recently, the interaction of FIN219 and FIP1 was particularly investigated (Chen et al., 2017). To extend the understanding of the regulatory mechanism between FR light signaling and the jasmonate (JA) response, Chen et al. (2017) determined the crystal structures of the FIN219-FIP1 complex with substrates. Furthermore, they showed that the interaction with FIP1 triggers enhanced activity of FIN219. According to their results, FIP1 (AtGSTU20) may regulate FIN219 activity, which further alters the level of JA signaling. Interestingly, the expression of tau GSTs, which were upregulated by methyl-JA treatment, was obviously higher than when treated with ethylene or salicylic acid (Wagner et al., 2002). The revealed structures of FIN219-FIP1 shed light on how FR light signaling may affect JA biosynthesis in order to regulate seedling photomorphogenesis in *Arabidopsis*. To define the functional role of *Arabidopsi*s GSTs in light-signaling pathways, Jiang et al. (2010) focused on several candidates affected by PHYA or FIN219. They performed dark-light transition experiments, where *AtGSTU17* expression depended strictly on PHYA. The phenotype examination of the *Atgstu17* mutant indicated that AtGSTU17 might have a function in the control of hypocotyl elongation in response to FR irradiation. Furthermore, the *AtGSTU17* overexpression line in the *phyA* mutant background revealed that this protein participates in the control of hypocotyl elongation, anthocyanin accumulation, FR blockage of greening, and flowering in a PHYA-dependent manner. Moreover, the expression pattern of *AtGSTU17* also appeared to be associated with auxin and abscisic acid (ABA) signaling and the GSH/GSSG ratio in the regulation of *Arabidopsis* development (Jiang et al., 2010). According to their results, AtGSTU17 protein is not only influenced by a PHYA-dependent pathway, but mediates the signaling and has a strong impact on the GSH/GSSG ratio, and thus on the redox status of the cells. Shohael et al. (2006) also found that light quality can influence the secondary metabolites and enzyme activities of somatic embryos grown in a bioreactor. The authors observed higher GST, but lower DHAR activity in *Eleutherococcus senticosus* somatic embryos affected by red or red and blue light. In contrast, blue light did not change significantly the activity of GST and DHAR in somatic embryos after 45 days (Shohael et al., 2006). Interestingly, it was found that, in red light, irradiated grapevine leaves, where the accumulation of resveratrol compound was enhanced (to protect grapevine from fungal pathogen, *Botrytis cinerea*), the expression of *GST* was rapidly upregulated and showed a peak after 12 h (Ahn et al., 2015). Yang et al. (2015) also revealed that red light induced resistance to *Pseudomonas syringae* pv. tomato DC3000 in tomato plants at night is associated with enhancement of GSH content and expression of *GST1*. In addition, red and blue light could effectively delay the symptom expression and replication of cucumber mosaic virus (CMV) in tobacco by increasing GSH content in the leaves (Chen et al., 2015). Interestingly, not only light quality but duration of the light application can determine the GSH content in leaves. High R/FR ratios induced accumulation of ascorbic acid and GSH content after 12 days in common bean (*Phaseolus vulgaris* L.) (Bartoli et al., 2009), but did not significantly changes the GSH levels after weeks in wheat (*Triticum aestivum* L.) leaves (Monostori et al., 2018). It can be concluded that pretreatment with artificial red light could alleviate the harmful effects of pathogens and abiotic stressors by increasing GST activity and expression, as well as GSH content in the leaves (Figure 1).

## GST Gene Expression and Enzyme Activity are Affected by Light Intensity

Light quality, as well as light intensity, has a great impact on the regulation of GST activity and gene expression. First of all, it was shown that darkness has a significant effect on GST activity and gene expression. In 2003, Dean et al. published a study in which the expression of the *GSTs* in darkness was determined in *Malva pusilla*. The main aim of the study was to identify *MpGST* genes connected to *Colletotrichum gloeosporioides* infection. According to their results, the transcript amount of some *GSTs* (*MpGSTZ1* and *MpGSTU2*) was induced as the infection developed, while *MpGSTF1* was induced during the transition from the biotrophic to the necrotrophic phase of the infection. They utilized dark pretreatment and found that the expression of both *MpGSTZ1* and *MpGSTU2* remained unchanged following transfer to the darkness, whereas the expression of *MpGSTU1* and *MpGSTF1* decreased by ~50 and 75%, respectively, when plants were placed in the dark for 2 h (Dean et al., 2003). However, Scalla and Roulet (2002) found that herbicide safener mefenpyr-diethyl treatment significantly increased GST activity and the expression of *HvGST6* (phi class) in dark-grown barley (*Hordeum vulgare* L. cv. Alexis) after 4 days.

Besides dark, low light and shade (reduced daylight) also influenced GST activity in plants. GST activity did not change under low light (60 μmol m^−2^ s^−1^) compared to controlled (160 μmol m^−2^ s^−1^) conditions in leaves and roots of micropropagated *Phalaenopsis* plantlet grown for 30 days (Ali et al., 2005). In contrast, GST activity declined under low (75 μmol m^−2^ s^−1^) and suboptimal light (225 μmol m^−2^ s^−1^) compared to controlled (400 μmol m^−2^ s^−1^) conditions in *Helianthus annuus* L. var. DRSF-113 seedlings after 72 h (Yadav et al., 2014). Similar changes were found in red leaf lettuce (*Lactuca sativa* L.) after 3 days in low light (40 μmol m^−2^ s^−1^), where the expression of *LsGST* (Unigene10814_All) significantly decreased compared to the control (100 μmol m^−2^ s^−1^) leaves (Zhang et al., 2018). GSH content also decreased upon low light in duckweed (*Lemna minor* L.) plants (Artetxe et al., 2002, 2006) and in *Arabidopsis* leaves (Oelze et al., 2011).

In contrast, high or excess light (2,500 μmol m^−2^ s^−1^) significantly elevated GST activity in *Arabidopsis* leaves (Mullineaux et al., 2000). A similar tendency was found by Ali et al. (2005) in leaves of micropropagated *Phalaenopsis* plantlet upon high light (300 μmol m^−2^ s^−1^). Moreover, based on gene expression data, high light (500 μmol m^−2^ s^−1^) stress caused a rapid induction of *PgGST* within 1 h in *Panax ginseng* (Kim et al., 2012). Lv et al. (2015) also observed that high light (1,200 μmol m^−2^ s^−1^) significantly increased the expression of *GST5* and *GST13* (tau class) and elevated GST activity in *Arabidopsis* leaves. Based on their result, β-cyclocitral (β-CC), a volatile oxidized derivative of β-carotene, can regulate NPR1 in order to promote GST transcription and subsequently increase GST activity in response to excess light. High light (1,000 μmol m^−2^ s^−1^) stimulated the elevation on GSH content in mustard (*Sinapis alba* L.) chloroplasts after 3 h (Baena-González et al., 2001) and in cashew plants (*Anacardium occidentale* L.) after 12 h upon high light (2,000 μmol m^−2^ s^−1^) (Lima et al., 2018). In contrast, high light (2,500 μmol m^−2^ s^−1^) decreased GSH content in Golden Agave (*Agave Americana* L.) leaves after 2 h (Deng, 2012) and in exocarp of apple (*Malus spp*.) after 3 h (Davey et al., 2004). However, there were not significant changes in GSH content after 4 days upon high light (600 μmol m^−2^ s^−1^) in cucumber (*Cucumis sativus* L.) leaves (Jiang et al., 2013). Interestingly, total GSH showed an initial increase during the first 30–40 min of high light (800 μmol m^−2^ s^−1^) treatment followed by a decrease (60 min) and an increase during dark recovery in two Antarctic lichens (*Usnea antarctica* Du Rietz) (Balarinová et al., 2014). Based on these results, increase in GST activity and GSH content was an adaptive response of the plants to higher amounts of ROS generated at higher light intensities. However, these changes were dependent on the light intensity, duration of irradiation and plant species or organs.

## Molecular Mechanism of Light Regulation of Plant GSTs

**Figure 2:**
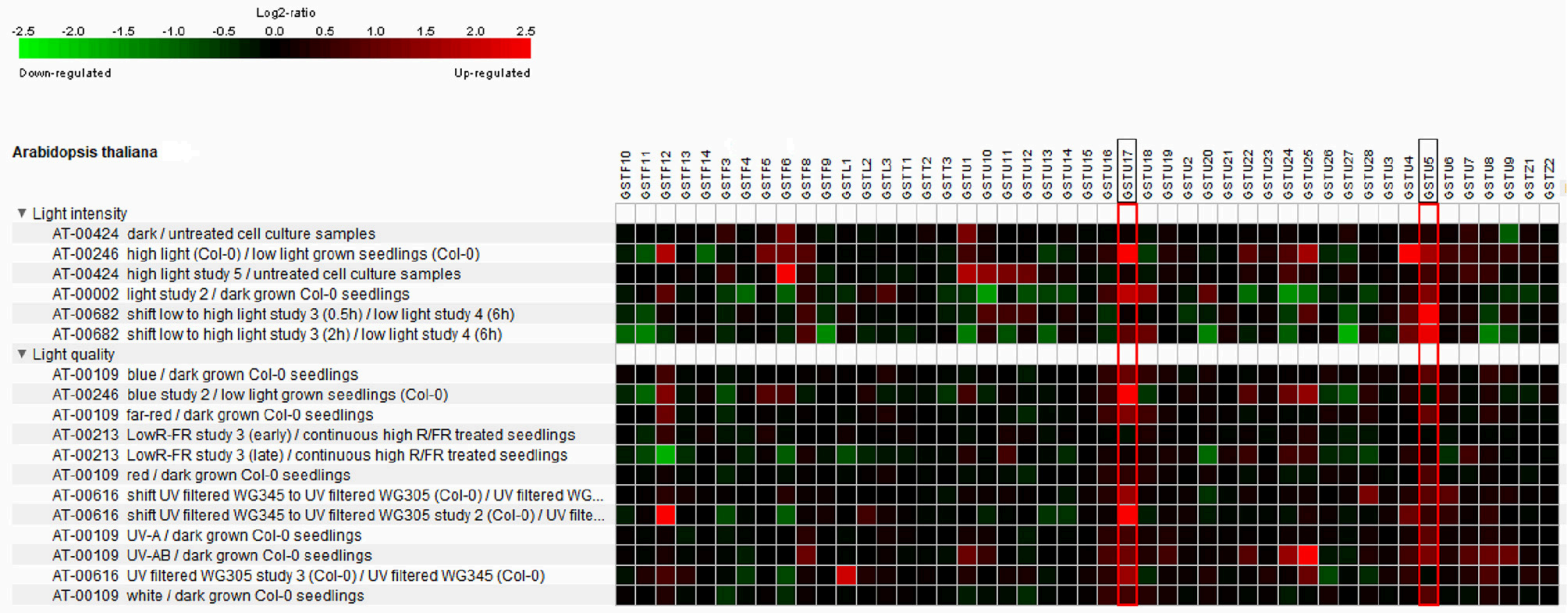
Heat map showing the light conditions as identified by Genevestigator which perturb *Arabidopsis* GST expression. The studies used in the analysis were *Arabidopsis* Col-0 seedlings with UV-A, UV-AB, white, blue, red far red light treatments compared to continuous dark (experiment ID: AT-00109 and AT-00002), the effect of low light-high light shift compared to low light control on detached rosette leaves of *Arabidopsis* (experiment ID: AT-00682), treatment of *Arabidopsis* cell cultures with high light and dark compared to untreated cell cultures (experiment ID: AT-00424), *Arabidopsis* Col-0 plant samples exposed to low red/far red compared to continuous high red/far red (experiment ID: AT-002013) and *Arabidopsis* Col-0 seedlings grown in filtered UV-B light conditions exposed to UV-B with a low UV-B filter for 6 h compared to continuously high and low UV-B (345 nm and 305 mn) filtered samples (experiment ID: AT-00616). Microarray data for all GSTs was used to construct heat map. Red indicates up-regulation, black no change, and green down-regulation with the color intensity reflecting the Log2 perturbation.

Regarding the functional overlaps and variability of GSTs, their expression and regulation show high diversity. Several microarray and transcriptome sequencing data confirm the effect of quality and quantity of light on the expression pattern of *GSTs*. *Arabidopsis GST* gene transcript data originating from Genevestigator (www.genevestigator.com, Hruz et al., 2008) are shown in Figure 2. The highlighted *GST* genes (*AtGSTU17* and *AtGSTU5*), were induced by most of the treatments, underlining their importance in light response and signaling. For example, the above-mentioned *AtGSTU17*, which participates in the signal transduction pathway of visible light, showed induction after almost every treatment. Besides *AtGSTU17, AtGSTU5* was similarly upregulated in most cases (Figure 2). Furthermore, UVB in several cases (AT-00616 and At-00109 datasets) induced the tau group *GST* expression except of some gene (e.g., *AtGSTU13* and *AtGSTU14*). White light, UV-B, red, and far red decreased the expression of some phi group sequences: *AtGSTF6, AtGSTF3*, and *AtGSTF11*. Downregulation of several *GST* genes (for instance *AtGSTF11, AtGSTU9, AtGSTU13*, and *AtGSTU27)* was seen after exposure to elevated light intensity.

To understand the processes other than gene expression changes in *GST* transcripts, it is necessary to collect the elements that are probably participating in the regulation. The 5′ *cis*-regulating elements (CRE) of *GST*s were described and categorized in several species, e.g., carnation (Itzhaki et al., 1994), soybean (Ulmasov et al., 1994, 1995), tobacco (Droog et al., 1995), *Arabidopsis* (Chen and Singh, 1999), Tausch's goatgrass (Xu et al., 2002), tomato (Csiszár et al., 2014), and pickleweed (Tiwari et al., 2016). Among the CREs, a great number of elements participates in the mediation of light signals. In *Arabidopsis*, among the upregulated GSTs, which were induced by different wavelength and light intensities, *AtGSTU17* and *AtGSTU5* share some common light-responsive elements (ATC-motif, Box 4, G-box, and LAMP–element).

*In silico* analysis of the 5′ regulatory region of 11 selected tomato *GSTs* revealed the presence of a high number of putative light-responsive elements in these genes (Csiszár et al., 2014). The CREs in the promoter regions of four *GSTs* with a light-responsive expression pattern were compared (Gallé et al., 2018), revealing that there was one common element (Box 4) in all four *GSTs*. Several G-box and Box I elements also presented in the promoters. Box 4 was described in the 5′ region of oat α-amylase. As it is a hyphenated palindrome sequence, it is likely to be a binding site of the helix-turn-helix and zinc finger classes of transcription factors (Rushton et al., 1992). Four *cis*-acting elements, designated as Boxes I, II, III, and IV, have previously been identified as functionally relevant components of the light-responsive *CHS* promoter in parsley (Weisshaar et al., 1991). Among them, Box I and Box II presented among the tomato *GST* CREs. These two elements are together called Unit 1 and necessary *cis*-acting elements for light response in the context of a minimal *CHS* promoter (Schulze-Lefert et al., 1989; Weisshaar et al., 1991). However, Unit 2 (Box III and Box IV), which enlarges the light responsiveness of Unit 1 is missing. The position of the two boxes differs from that in parsley *CHS*, as in most cases they are further than −600.

## Conclusion and Perspectives

Light intensity and quality are the major factors limiting photosynthesis, in turn affecting carbohydrate production and eventually plant growth and development as well as defense reaction (Chen et al., 2004). It has been suggested that red and blue light or both low- (shade) and high-light intensities can influence the fitness of plants (Su et al., 2017). Moreover, light regulates the activity and gene expression of *GSTs*, which are key elements of detoxification. Two classes of GSTs, tau and phi, play pivotal role in the detoxification of the effects of herbicides (Dixon et al., 2003). The effectiveness of some commonly used photo-inhibitor herbicide compounds, such as terbuthylazine or metribuzin, depends on the photosynthetic electron transport, meaning they are light-dependent. A majority of herbicides are detoxified through substitution reactions and, on a much rarer basis, GSH addition reactions (Cummins et al., 2011; Chronopoulou et al., 2017). In this way, GSTs are involved in desired traits of herbicide tolerance or resistance, e.g., in crops or weeds, respectively (Chronopoulou et al., 2017). Moreover, candidates of tau and phi groups of GSTs were found to play roles in altering the capacity of crops to metabolize herbicides and other xenobiotics; thus, they are important components of safener effects (Brazier-Hicks et al., 2018). Detailed information about the transcriptional inductions of these detoxifying enzymes has been a valuable addition to safener innovation in agriculture. Light regulation of these processes may interact, strengthen or weaken safener-induced enhancement in detoxification efficiency, thereby offering the possibility to reduce pesticide usage.

Circadian regulation in several plant species revealed some similarities: the activity and expression levels of GSTs reached the maximum at the end of the light period before both decreased under darkness. Thus, GSTs seem to be regulated by light, while their participation in light-dependent cellular mechanisms is complex: some of them were found to be a transducer of the UV- and red light-regulated signaling pathways. Processes behind the light-induced switch of GSTs are often altered by the intensity, duration and quality of the illumination, where the wavelength seems to be the most emphasized parameter. Especially red light, when it was applied as a pretreatment, was proven to be so effective that it could even alleviate the effect of biotic stressors by increasing GST activity. UV-B radiation in combination with herbicides may also enhance oxidative stress and decrease glutathione-mediated detoxification in weeds, causing severe damages to lipids and proteins and, in turn, decreasing membrane stability and inducing cell death. However, both light quality as well as light intensity influences GST activity and gene transcription. Darkness, low light or shade mostly reduced GST activity, while high light significantly elevated the activity and expression of GSTs and also GSH levels. Nevertheless, these changes are not only dependent on the light intensity, but also on the duration of the illumination and plant species and organs, respectively. The light-dependent regulation of plant *GST* expression was also confirmed by *in silico* promoter analysis. The presence of a high number of light-responsive elements also indicates that light plays an important role in the regulation of GST activity and gene expression. However, further research on plant species (crops and weeds) or organs and temporal regulation of GST activity and gene expression is necessary for understanding their complex regulation under various light conditions. Moreover, the crosstalk among other detoxifying enzymes and other signaling compounds under different light conditions is also worthy of further investigation.

As a summary, light responsiveness seems to be a constant and permanent feature of GSTs, which determines the detoxification, adaptation, stress responses, and even their reaction to dark. Understanding the mechanism that can regulate plant GSTs activity and gene expression at molecular and physiological levels is a major problem in current plant biology as well as in agriculture. Based on this knowledge, integrating the application time of spraying herbicides or safeners (in the light or dark period) with the knowledge of plant detoxification processes by GSTs into weed and pest management programs can reduce agricultural costs and increase the effectiveness of crop protection.

## Author Contributions

ÁG and PP conceived of the presented idea. ÁG and PP were involved in planning and drafted the manuscript. JC supervised the work. KB designed the table. EH performed the promoter test. AÖ and ZC aided in interpreting the results. ÁG, PP, and JC worked on the manuscript. All authors discussed the results and commented on the manuscript.

### Conflict of Interest Statement

The authors declare that the research was conducted in the absence of any commercial or financial relationships that could be construed as a potential conflict of interest.
